# *ACE* Insertion/Deletion Polymorphism (rs4646994) Is Associated With the Increased Risk of Multiple Myeloma

**DOI:** 10.3389/fonc.2019.00044

**Published:** 2019-02-06

**Authors:** Szymon Zmorzynski, Aneta Szudy-Szczyrek, Sylwia Popek-Marciniec, Iwona Korszen-Pilecka, Magdalena Wojcierowska-Litwin, Małgorzata Luterek, Sylwia Chocholska, Wojciech Styk, Grazyna Swiderska-Kołacz, Joanna Januszewska, Michal Mielnik, Marek Hus, Agata A. Filip

**Affiliations:** ^1^Department of Cancer Genetics with Cytogenetic Laboratory, Medical University of Lublin, Lublin, Poland; ^2^Chair and Department of Hematooncology and Bone Marrow Transplantation, Medical University of Lublin, Lublin, Poland; ^3^Department of Animals Physiology, The Jan Kochanowski University in Kielce, Kielce, Poland; ^4^The Regional Blood Donors Center of Kielce, Kielce, Poland

**Keywords:** *ACE* gene, I/D polymorphism, plasma cell myeloma, bortezomib, rs4646994

## Abstract

**Introduction:** The insertion (I allele) deletion (D allele) polymorphism of *ACE* gene (rs4646994) may influence the etiopathogenesis of multiple myeloma (MM). *ACE* gene is expressed in bone marrow cells and encodes angiotensin converting enzyme (ACE). It converts angiotensin I to active peptide angiotensin II, which stimulates proliferation of hematopoietic stem cells. This suggests possible association of *ACE* I/D gene polymorphism with MM. The aim of our study was to check possible impact of this polymorphism on risk of development and outcome of MM, as well as, sensitivity to bortezomib in cell cultures derived from MM patients.

**Objects and Methods:** Genomic DNA from 98 newly diagnosed MM patients and 100 healthy blood donors were analyzed by PCR method. Chromosomal aberrations were detected by use of cIg-FISH. In a subgroup of 40 MM patients nucleated bone marrow cells were treated with bortezomib *in vitro*.

**Results:** The Hardy-Weinberg equilibrium test showed that genotypic frequencies diverged significantly from the equilibrium. The differences between I and D allele frequencies in control and study population were significant (*p* = 0.046). We observed the association between DD genotype and more than 2-fold risk of MM - OR = 2.69; *p* < 0.0001. We did not detect any significant differences among studied genotypes regarding clinical and laboratory parameters. Moreover, we did not observe the association between survival of MM patients and I/D genotypes. Bortezomib increased number of apoptotic and necrotic cells, but the only statistically significant differences were observed in the number of viable cells at 1 nM between ID and DD genotypes (*p* = 0.026).

**Conclusion:** Presented results confirmed the significant relationship between *ACE* (I/D) polymorphism and risk of MM development. We did not observe the association of *ACE* I/D polymorphism with disease outcome and bortezomib *in vitro* sensitivity.

## Introduction

Multiple myeloma (MM) is characterized by the proliferation of malignant, clonal B-lymphocytic cells in bone marrow (1). The symptoms that appear in the course of disease include anemia, bone damage, hypercalcemia and also renal dysfunction (2, 3). Kidney disease is a common complication of MM, which occurs in 20–25% patients at diagnosis and in up to 50% patients during the course of disease (3, 4). In MM patients persistent kidney dysfunction is most commonly caused by tubular nephropathy due to secreted monoclonal immunoglobulin or monoclonal light chain (5).

Changes occurring in the bone marrow microenvironment lead to transformation of normal B lymphocytes into the malignant cells (6). Cancer cells are characterized by an increased proliferation and the ability to metastasize. These processes are regulated in part by the ubiquitin-proteasome system (UPS) (7). Bortezomib is a proteasome inhibitor approved for clinical use in MM patients and its function affects intracellular protein degradation. The inhibition of proteasome causes many effects, for example apoptosis of bone marrow cells (8, 9).

Bone marrow renin-angiotensin-aldosterone system (RAAS) influences the function of transcriptional factors and the response to growth factors released by microenvironment. A local bone marrow RAAS can affect proliferation of physiological and malignant cells (10). RAAS affects tumor growth and metastasis by modulating many processes such as proliferation of bone marrow cells (11). The RAAS includes the angiotensin I converting enzyme (ACE), which may be associated with increased cell proliferation (12). The main mechanism responsible for that function is cleavage by ACE enzyme of proteins, which show anti-proliferative effect on bone marrow cells (13).

Genetic factors play a major role in the pathogenesis of hematological malignancies including multiple myeloma. The *ACE* gene (17q23.3 *locus*) consists of 26 exons and 25 introns, and codes ACE enzyme (14). Functional polymorphism is present in intron 16 in the form of insertion (I allele) and/or deletion (D allele) of 289 bp *Alu* repeat sequence (rs4646994) (15). The I/D polymorphism may affect the expression of *ACE* gene and/or the function of angiotensin I converting enzyme (16). The DD genotype is associated with vessel wall thickness and higher blood pressure (17). The presence of D allele is associated with higher ACE enzyme activity and higher production of angiotensin II in comparison to I allele (18). Angiotensin II is known to activate several signaling pathways, including mitogen-activated protein kinase (MAPK), phosphoinositide-3-kinase (PI3K)/AKT and protein kinase cAMP-dependent pathways, which play a role in regulation of cell growth, differentiation, reorganization of cytoplasmatic proteins and cell cycle progression (19). More and more data indicate that *ACE* gene product may be involved in cancer development (20). However, little is known on the biological and clinical significance of *ACE* I/D polymorphism in the context of MM.

The aim of our study was the analysis of correlation between *ACE* I/D polymorphism with the risk of development and the course of MM. Furthermore, we have analyzed whether this polymorphism predicts sensitivity to bortezomib in cell cultures derived from studied patients.

## Materials and Methods

### Patients and Samples

For the study, bone marrow aspirates and peripheral blood samples were collected from 98 newly-diagnosed patients with MM, who were hospitalized at the Chair and Department of Hematooncology and Bone Marrow Transplantation, Medical University of Lublin in years 2013–2018. The study was conducted after obtaining a positive opinion from the Bioethics Committee (no. KE-0254/165/2013 and no. KE-0254/337/2016), according with the ethical standards established by Helsinki Declaration. The research material was collected upon all patients and healthy blood donors provided written informed consent.

The characteristics of MM patients is shown in Table 1.

**Table 1:** The characteristics at diagnosis of MM patients included to the study.

**Patients**	**All patients *n* = 98**	**II homozygous *n* = 13**	**Heterozygous *n* = 27**	**DD homozygous *n* = 58**
Male/female	52/46	6/4	16/12	30/30
Mean age (years) at diagnosis	65.6	65.75	65.3	65.88
**TYPE OF MM**				
IgG, *n* (%)	55 (56.12)	4	15	36
IgA, *n* (%)	25 (25.51)	4	8	13
Light chain, *n* (%)	17 (17.34)	2	4	11
Free light chain ratio	303	166	253	356
Non-secretory, *n* (%)	1 (1.02)	0	0	2
**STAGE ACCORDING TO THE INTERNATIONAL STAGING SYSTEM**, ***n*** **(%)**				
I, *n* (%)	26 (26.5)	3	10	13
II, *n* (%)	30 (30.61)	4	9	17
III, *n* (%)	42 (42.85)	2	8	32
No renal failure, *n* (%)	80 (81.63)	8	24	48
Renal failure, *n* (%)	18 (16.32)	2	3	13
**THE STAGE OF KIDNEY DISEASE**				
G1, *n* (%)	31 (31.6)	0	12	19
G2, *n* (%)	25 (25.51)	5	7	13
G3A, *n* (%)	15 (15.3)	2	1	12
G3B, *n* (%)	12 (12.24)	2	4	6
G4, *n* (%)	6 (6.12)	0	2	4
G5, *n* (%)	9 (9.18)	1	1	7
Percentage of plasma cells in bone marrow, M ± SD	30.54 ± 18.96	29.17 ± 19.54	30.67 ± 19.8	29.15 ± 18.43
**CYTOGENETIC CHANGES**				
del(17p13.1)	3	0	1	2
t(4;14)	8	1	3	4
t(14;16)	1	0	0	1
Albumins (g/dL) M ± SD	3.56 ± 0.67	3.63 ± 0.67	3.56 ± 0.67	3.55 ± 0.67
β2-microglobulin (mg/L), M ± SD	5.98 ± 3.98	6.37 ± 4.14	6.02 ± 3.99	6.01 ± 3.98
Calcium (mM/L) M ± SD	2.43 ± 0.3	2.45 ± 0.32	2.43 ± 0.3	2.43 ± 0.3
Hemoglobin (g/dL) M ± SD	10.37 ± 1.91	10.28 ± 1.85	10.36 ± 1.89	10.33 ± 1.85
Creatinine (mg/dL) M ± SD	1.55 ± 1.67	1.67 ± 1.78	1.55 ± 1.68	1.56 ± 1.68
C-reactive protein (mg/L), M ± SD	16.07 ± 35.34	16.73 ± 37.43	16.27 ± 35.72	16.23 ± 35.51
Progression free survival^*^ (PFS) (months), M ± SD	17.78 ± 18.18	18.72 ± 19.07	17.7 ± 18.15	17.78 ± 18.18
Death caused by MM, n (%)	31 (31.63)	6	5	20
Overall survival (months) M ± SD	25.21 ± 26.31	26.79 ± 26.81	25.11 ± 26.51	25.41 ± 26.37

*SD, standard deviation; M, mean*.

* *The time elapsed between treatment initiation and tumor progression or death from any cause (21)*.

Therapeutic regimens consisted of thalidomide combined with steroids and/or chemotherapy followed or not by autologous hematopoietic stem cell transplant (auto-HSCT). Response was evaluated at the end of treatment using the International Myeloma Working Group guidelines, and classified as stringent complete remission (sCR), complete response (CR), very good partial response (VGPR), partial response (PR), minimal response (MR), stable disease (SD) or progressive disease (PD) as described elsewhere (22, 23). Overall survival (OS) encompassed time from diagnosis until relapse, progression, death due to tumor effect or last follow-up, and time from diagnosis until death by any cause or last follow-up, respectively. The median follow-up time of MM patients enrolled in the study was 18 months (range 1–100 months).

Peripheral blood was used for DNA isolation and I/D polymorphism determination of *ACE* gene. From bone marrow aspirates, cell cultures were established to carry out the research associated with cIg-FISH (*n* = 98) and bortezomib treatment (*n* = 40).

Control samples were made of peripheral blood obtained from 100 healthy blood donors attending the Regional Blood Donation and Blood Treatment Center in Kielce.

### DNA Isolation

DNA isolation from peripheral blood was performed using a commercial kit (Qiagen, Germany) according to manufacturer's procedure. The concentration and quality of DNA was checked using NanoDrop device (Thermo Fisher Scientific, USA).

### *ACE* Gene Polymorphisms—Genotyping

The PCR method with two primers according to Yoshida (with modifications) was applied for analysis of *ACE* polymorphism (24). *ACE* gene intron 16 fragment length of 490 bp was amplified by using primers:

-forward 5′-CTG GAG ACC ACT CCC ATC CTT TCT-3′
-reverse 5′-GAT GTG GCC ATC ACA TTC GTC AGA T-3′

Each PCR mixture (25 μl) contained 150 ng genomic DNA and PCR buffer (Clontech Laboratories, USA), dNTPs mixture (0, 25 mM), HD polymerase (Clontech Laboratories, USA) and primers (10 μM of each). The mixture was heated 94°C for 5 min and underwent 35 cycles of amplification: denaturation 94°C for 30 s, annealing 58°C for 45 s, elongation 72°C for 30 s. The final elongation took 2 min at 72°C. The PCR reaction was performed in a Applied Biosystems 9700 Thermal Cycler.

**Figure 1:**
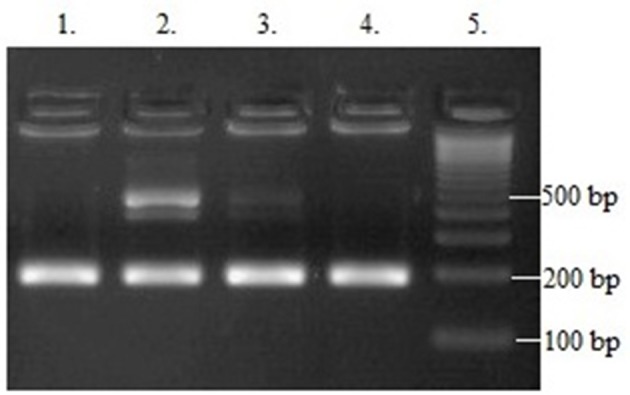
Results of *ACE* (I/D) gene polymorphism analysis: Line 1 and 4−190 bp band (DD homozygote); Line 2 and 3−190 and 490 bp bands (ID heterozygote); Line 5—leader marker (100 bp).

The PCR product was analyzed on 3% agarose gel and stained with SimplySafe (Eurx, Poland) and visualized in G:Box (Syngene, Great Britain) (Figure 1). D- or I-alleles were identified by the presence of 190 or 490 bp fragments, respectively. Heterozygous ID genotype shows the presence of two bands at 490 bp and one band at 190 bp. An independent PCR analysis was carried out for each sample.

### Cytogenetic Analyses

**Figure 2:**
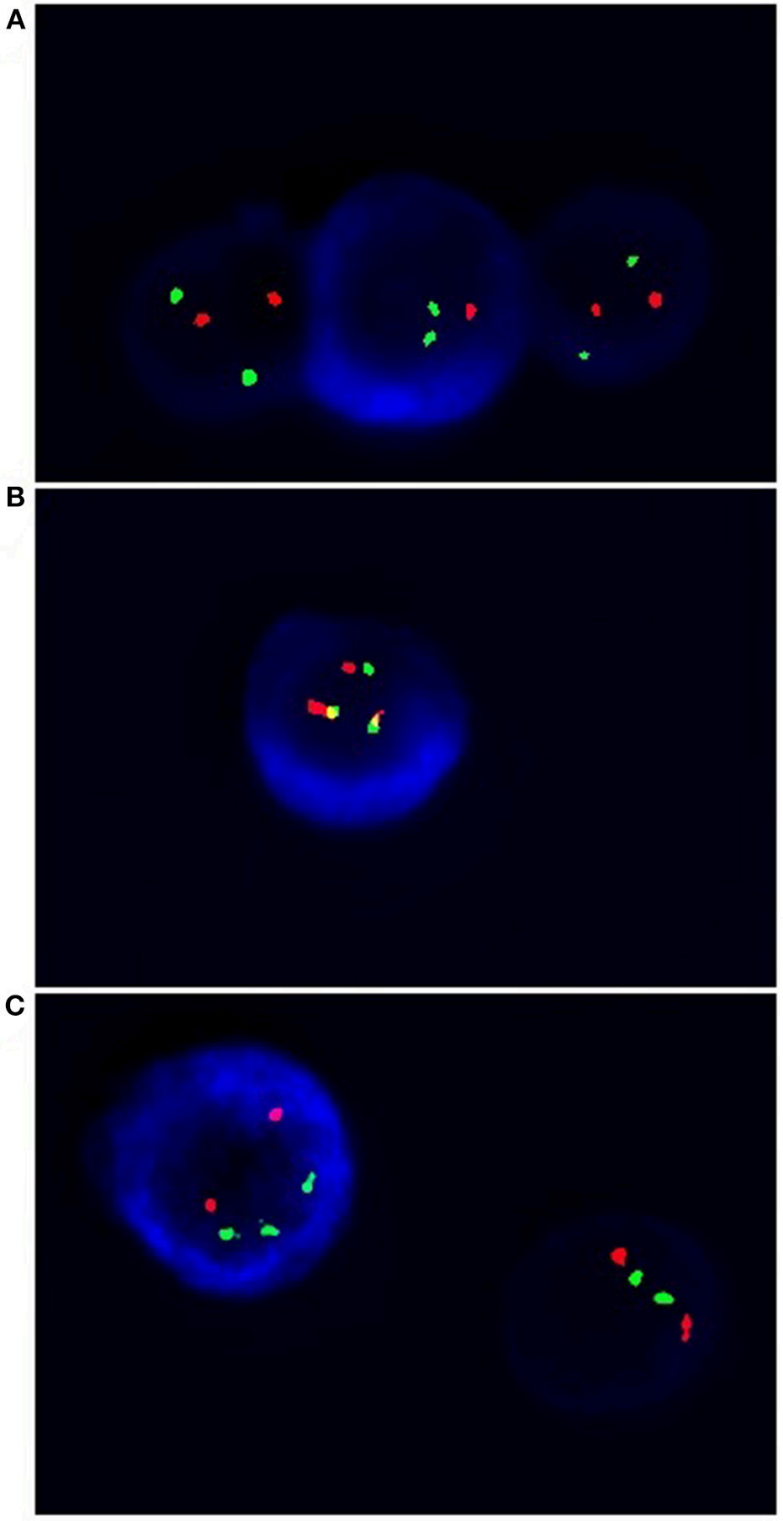
Simultaneous staining of cytoplasmic immunoglobin and FISH (cIg-FISH). The following probes were used: **(A)** Vysis TP53 (red signals)/CEP 17 (green signals) for detection of del(17p13.1), monoallelic deletion; **(B)** Vysis IGH (green signals)/FGFR3 (red signals) for detection of t(4;14)(p16;q32) (yellow signals); **(C)** Vysis IGH (green signals)/MAF (red signals) for detection of t(14;16)(q32;q23). Total magnification of 1.500 ×.

Abnormalities essential for MM, such as del(17p13.1) and *IgHV* gene rearrangements—t(4;14), t(14;16) were tested by cIg-FISH according to Ross et al. (25) recommendations (25). Cultured bone marrow malignant plasma cells from 98 patients were identified using simultaneous staining of cytoplasmic immunoglobulin and FISH (cIg-FISH) according to the previously described protocol with modifications (26, 27). The following probes, all from Abbott Molecular (Abbott Park, IL, USA), were used: Vysis TP53/CEP 17 FISH Probe Kit for detection of del(17p13.1), Vysis IGH/FGFR3 DF FISH Probe Kit for detection of t(4;14)(p16;q32), and Vysis IGH/MAF DF FISH Probe Kit for detection of t(14;16)(q32;q23) (Figure 2). Fluorescent microscopic analysis was performed by scoring 100 AMCA-positive plasma cells to determine the frequency of each aberration. Cut off level were 20% for deletion probes and 10% for dual fusion probes, according to the recommendations of the European Myeloma Network (25).

### Cell Cultures, Apoptosis and Necrosis Detection

**Figure 3:**
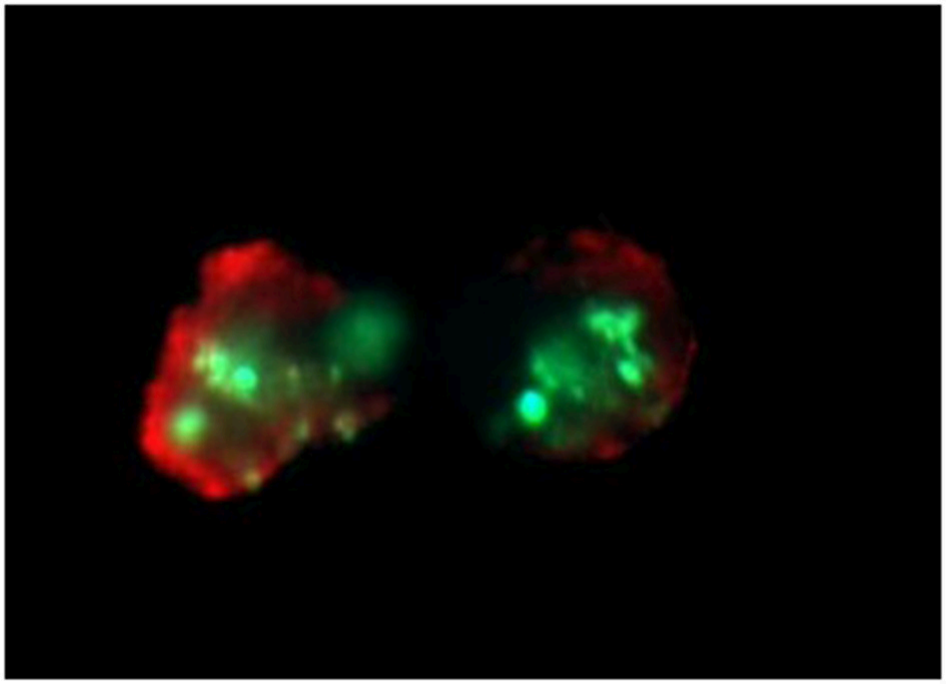
Example of *in vitro* bortezomib activity (at 2 nM). Below are shown apoptotic cells, which are stained both AnnCy3 (red) and 6-CF (green). For statistical analysis of necrotic/apoptotic/viable cells plasmocytes with diameter 9–12 μm were counted. Cells were stained by immunofluorescent technique as described in Material and methods. Total magnification of 1.500 ×.

Bone marrow aspirates (*n* = 40) (mean number of plasma cells - 34% ± 17) were stratified on Lymphoprep (Axis-Shield PoC As, Norway) and lymphocyte fraction was used to established cell cultures, which were grown in 15 ml of culture medium—RPMI 1640 with L-glutamine (Biomed, Poland); 10% inactivated fetal calf serum (Biomed, Poland), 1% antibiotic antimycotic (A&E Scientific, Belgium), and different doses of bortezomib (LC Laboratories, USA, 200 mg/ml) - 1 nM/2 nM/4 nM. In the subgroup of 20 patients a higher bortezomib concentration (8 and 12 nM) was applied (data not shown) to estimate the 50%-cell growth inhibition. The dose 12 nM of bortezomib caused the above-mentioned inhibition. Bortezomib was dissolved in DMSO and stored at −80°C. The final DMSO concentration in culture medium was < 0.1%. As a control cell cultures without bortezomib (with 0.1% DMSO) were used. The amount of 1–1.5 ml of lymphocyte fraction (from each patient) was added, respectively, to the 15 ml of culture medium. The cultures were grown at 37°C in the atmosphere of 5% CO_2_ for 24 h (without G-CSF). The cell cultures were routinely terminated and cell suspensions were prepared to determine the number of apoptotic, necrotic and viable cells by means of Annexin V-Cy3 Apoptosis Detection Kit according to manufacturer's protocol (Sigma-Aldrich, USA). For fluorescence microscopy viable cells were stained with 6-CF (6-carboxyfluorescein)—green, necrotic cells were stained only with AnnCy3 (Annexin V Cy3.18). Cells starting apoptotic process were stained both with AnnCy3 (red) and 6-CF (green) (Figure 3). Mainly plasma cells (with diameter 9-12 μm) were analyzed using fluorescence microscope according to Carter's et al. (28).

### Statistical Analysis

Laboratory values of MM patients with polymorphism were compared using an independent *t*-test for continuous variables and Chi-square test for categorical variables. The association of *ACE* I/D polymorphism with prognostic factors was evaluated using Chi-square test or Fischer's exact test. The quantitative data was shown as frequency or percentage. Deviation of genotype frequencies in controls and cases from Hardy-Weinberg equilibrium (HWE) was assessed by Chi-squared test with one degree of freedom (*df*) using the Michael H. Court's (2005–2008) calculator (29). For 95% confidence interval (CI), we assumed *p* = 0.05 and χ^2^ = 3.84; therefore, if the χ^2^ = ≤ 3.84 and the corresponding *p* ≥ 0.05 then the population is in HWE. The Cox proportional hazard model was used for univariate and multivariate analysis of time-to-event and OS. The ANOVA test was used to analyze OS and PFS including different schemes of treatment and presence or absence of ASCT. The Kaplan-Meier method and the log-rank test were used for survival analysis. We assumed a 5% error of inference and the related level of significance *p* < 0.05 pointing to the existence of statistically significant differences. Statistical analyzes were performed using the Statistica ver. 12.5 (StatSoft).

## Results

Fifty-Two males (53%) and 46 females (47%) among 98 MM patients were included in the study, with median age of 65.6 years. Detailed clinical characteristics at diagnosis are listed in Table 1. Genotyping was successful in all individuals investigated within the study. The HWE test confirmed that the genotypic frequencies for healthy individuals (controls) were balanced, while for MM patients diverged significantly from the equilibrium, thereby indicating possible disease association (Table 2). The differences between allelic frequencies in control and study groups were statistically significant. Allele frequencies in patient and control populations are summarized in Table 3. The frequency of D allele was higher, than the I allele, in both populations. Moreover, an association between MM and DD genotype was observed; OR = 2.69; *p* < 0.0001. The presence of I allele in heterozygotes did not elevate the MM risk - OR 0.41; *p* = 0.003. The associations between *ACE* I/D genotypes and MM risk are presented in Table 4.

**Table 2:** Hardy-Weinberg equilibrium for *ACE* (I/D) polymorphism in case and control groups according to expected (E) and observed (O) values.

	**II**	**ID**	**DD**	**Total**	**HWE *p*-value and **χ**^2^**
**CONTROL**					
E	14.4	47.1	38.4	100	0.85
O	14	48	38	100	0.03
**CASE**					
E	5.6	35.7	56.6	98	**0.014**
O	10	27	61	98	**5.91**

*Bold values indicate statistical significant*.

**Table 3:** The comparison of *ACE* I/D allele frequencies among Polish Caucasian MM patients and controls.

***ACE* gene alleles**	**MM cases (*n* = 98) *n* (%)**	**Controls (*n* = 100) *n* (%)**	***p*-values**
I	47 (24)	66 (33)	–
D	149 (76)	134 (67)	**0.046**
Total	196 (100)	200 (100)	–

*Bold values indicate statistical significant*.

**Table 4:** Association between *ACE* I/D genotypes and risk of MM.

**Genotypes**	**MM cases *n* (%)**	**Controls *n* (%)**	**OR (95% CI)**	***p-*value**
II	10 (10.2)	14 (14)	1	–
ID	27 (27.55)	48 (48)	0.78 (0.31–2.01)	0.61
DD	61 (62.24)	38 (38)	2.25 (0.91–5.57)	0.076
Total	98 (100)	100 (100)	–	–
ID and II	37	62	1	–
DD	61	38	**2.69** (1.52–4.78)	**< 0.0001**
Total	98 (100)	100 (100)	–	–
II and DD	71	52	1	–
ID	27	48	**0.41** (0.23–0.74)	**0.003**
Total	98 (100)	100 (100)	–	–
ID+DD	88	86	1	–
II	10	14	0.7 (0.29–1.66)	0.41
Total	98 (100)	100 (100)	–	–

*Bold values indicate statistical significant*.

Furthermore, we analyzed potential relation between clinical and laboratory results at diagnosis of MM patients and I/D genotypes. We did not confirm any significant differences among studied genotypes regarding such parameters as sex, age of MM onset, ISS stage, cytogenetic changes, type of MM (secretory-, non-secretory MM, light chain disease), monoclonal protein class (IgG, IgA, etc.), light chain type (kappa, lambda), loss of body weight before treatment, baseline hemoglobin concentration, estimated glomerular filtration rate, the presence of renal insufficiency at diagnosis of MM, levels of albumin, β2-microglobulin, calcium, creatinine, and C-reactive protein.

**Figure 4:**
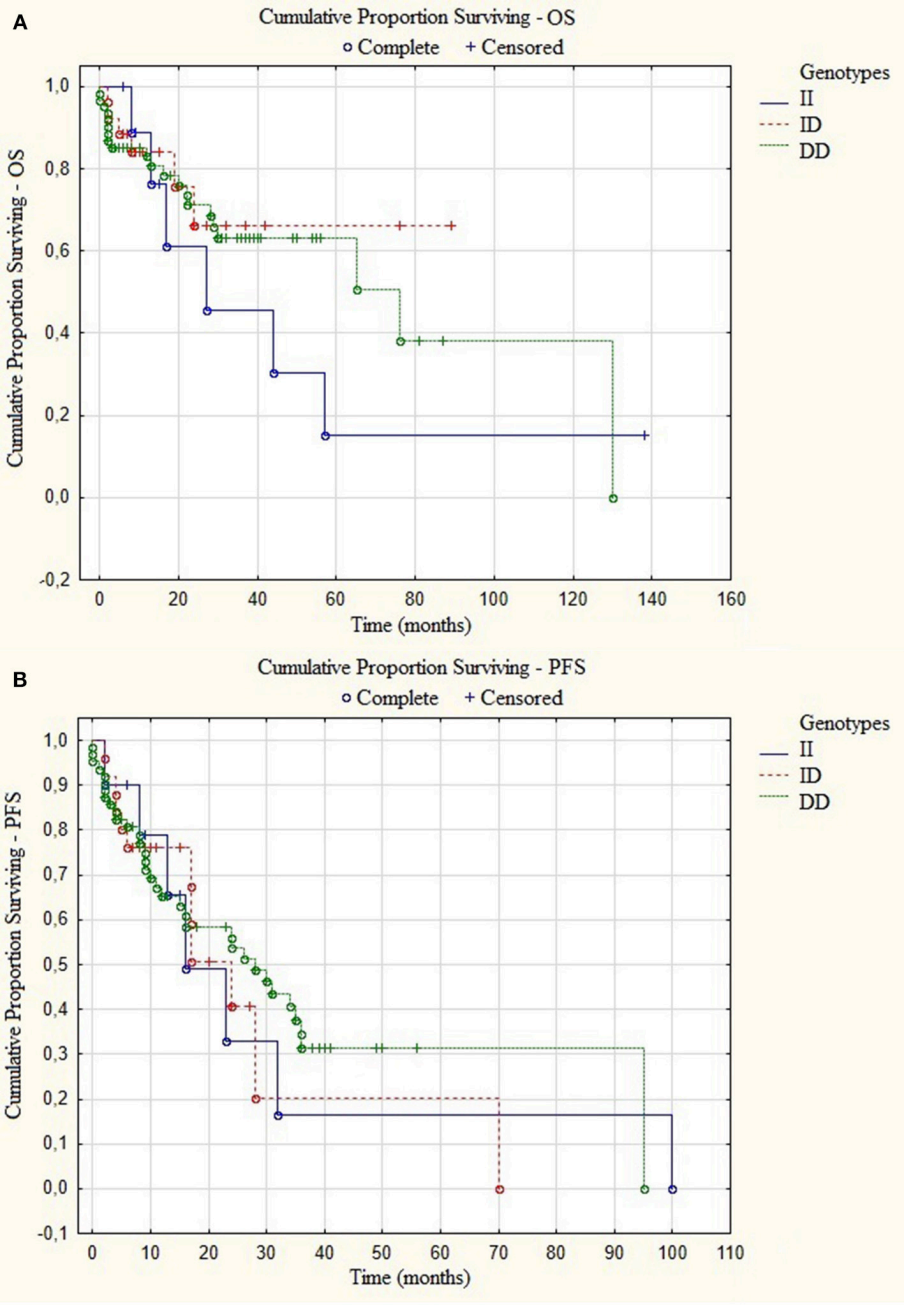
Kaplan-Meier analysis of **(A)** overall survival (OS) *p* = 0.94 and **(B)** progression-free survival (PFS) *p* = 0.99 in group of MM patients with different I/D genotypes.

An univariate Cox analysis revealed that patients at stage III of ISS and patients who did not receive auto-HSCT had 2.79 and 4.36 increased risk of disease relapse or progression, respectively (Tables 5, 6). The multivariate regression analysis also confirmed that patients without auto-HSCT had statistically higher risk (*H* = 4.62) of disease progression (Table 5). We did not find the correlation between survival of MM patients and I/D genotypes (Figure 4). Time-to-event analysis, where the event was age at diagnosis, showed that DD, ID and II genotypes did not play a role for disease onset (*p* ≥ 0.35).

**Table 5:** *ACE* I/D polymorphism in survival rate of MM patients.

**Variable**	**Univariate Cox analysis**			**Multivariate Cox analysis**		
	***p*-value**	**HR**	**95% CI**	***p*-value**	**HR**	**95% CI**
**ISS**						
I+II	–	Reference	–	–	Reference	–
III	**0.005**	**2.79**	1.36–5.73	0.08	3.05	(−1.48)−0.08
**AUTO-HSCT**						
Yes	–	Reference	–	–	Reference	–
No	**0.002**	**4.36**	1.70–11.17	**0.03**	**4.62**	−2.11−(−0.09)
**ACE I/D**						
II+ID	–	Reference	–	–	Reference	–
DD	0.83	0.93	0.45–1.89	0.96	0.002	(−0.90)−0.95
**ACE I/D**						
ID+DD	0.32	0.63	0.26–1.57	0.54	0.36	(−0.79)−1.50
II	–	Reference	–	–	Reference	–

*Bold values indicate statistical significant*.

**Table 6:** *ACE* I/D polymorphism in response rate of MM patients.

**Variable**	**Response rate**	
	**CR + VGPR + PR**	**SD + PD**
	***p*-value**	**OR (95% CI)**
**ISS**		
I+II	–	Reference
III	**0.03**	**0.31 (0.1–0.92)**
**AUTO-HSCT**		
Yes	–	Reference
No	0.34	0.59 (0.2–1.75)
**ACE I/D**		
II+ID	–	Reference
DD	0.25	0.52 (0.17–1.62)
**ACE I/D**		
ID+DD	–	Reference
II	0.34	2.57 (0.3–22)

*CR, complete response; VGPR, very good partial response; PR, partial response; SD, stable disease; PD, progressive disease patients—according to response criteria for MM (22, 23). Bold values indicate statistical significant*.

The survival analysis showed that MM patients without ASCT and with II genotype treated with thalidomide have longer OS (54 months, *p* = 0.03) in comparison to MM patients treated with bortezomib (OS = 27 months) and both—thalidomide and bortezomib (OS = 21 months) (Table 7). In the case of PFS analysis the results were statistically insignificant.

**Table 7:** Survival analysis of MM patients including different treatment and auto-HSCT.

**Genotypes**	**Patients with auto-HSCT treated with**				**Patients without auto-HSCT treated with**			
	**T**	**B**	**M**	***p*-value**	**T**	**B**	**M**	***p*-value**
**OS (MONTHS)**								
II+ID	22	11.6	9.6	0.09	50.6	35	23	0.08
DD	23.5	21	6	0.31	86.2	19	18.3	0.07
ID+DD	21.2	18.4	8.1	0.18	^*^	^*^	^*^	^*^
II	32.7	14	9	0.27	**53.9**	**27**	**21**	**0.03**
**PFS (MONTHS)**								
II+ID	29.3	35	23	0.73	13.1	10.4	9.6	0.60
DD	49.5	17	18.3	0.48	19.5	13.6	5.6	0.27
ID+DD	^*^	^*^	^*^	^*^	17	12.8	8	0.24
II	30	26	21	0.53	17.5	11.2	9	0.61

*T, Thalidomide; B, bortezomib; M, Thalidomide and Bortezomib*.

* *Too small group for analysis. Bold values indicate statistical significant*.

Bortezomib increased number of apoptotic and necrotic cells in all studied genotypes. The only statistically significant difference was observed in the number of viable cells at 1 nM between ID and DD genotypes (*p* = 0.026; 82.15 vs. 74.74%). More detailed data are shown in Table 8. The dose of 12 nM (not shown) caused death of at least 50% of cells in all genotypes.

**Table 8:** The effect of different bortezomib doses on bone marrow cells apoptosis, viability and necrosis.

**Genotypes**	**Bortezomib/DMSO**			
	**Control 0.1% DMSO (0 nM)**	**1 nM**	**2 nM**	**4 nM**
**APOPTOTIC CELLS (%)**				
II	4.49	9.80	14.12	20.18
M(±SD)	(5.22)	(12.38)	(7.01)	(11.24)
ID	5.27	14.06	18.01	24.96
M(±SD)	(5.31)	(12.58)	(6.92)	(11.50)
DD	6.53	20.55	17.05	26.74
M(±SD)	(5.19)	(12.73)	(7.16)	(9.76)
**NECROTIC CELLS (%)**				
II	1.93	3.65	5.84	11.26
M(±SD)	(3.67)	(3.04)	(4.54)	(6.62)
ID	2.01	5.16	5.50	12.01
M(±SD)	(3.75)	(3.09)	(4.66)	(6.71)
DD	3.57	4.70	7.37	11.53
M(±SD)	(3.76)	(3.15)	(4.59)	(6.79)
**VIABLE CELLS (%)**				
II	90.54	73.76	67.04	61.69
M(±SD)	(7.43)	(12.41)	(12.70)	(12.82)
ID	90.64	82.15^*^	77.92	66.76
M(±SD)	(7.61)	**(12.65)**	(11.74)	(13.12)
DD	87.67	74.74^*^	75.33	64.12
M(±SD)	(7.71)	**(12.76)**	(11.16)	(12.51)

*Below are showed average values. M, mean; SD, standard deviation*.

* *differences statistically significant, p = 0.026. Bold values indicate statistical significant*.

We have found the significant relationship between DD genotype and risk of MM development. We did not observe the association of *ACE* I/D polymorphism with disease outcome and bortezomib *in vitro* sensitivity.

## Discussion

In this study, we have explored the association of *ACE* I/D polymorphism with MM in Caucasian population. To our knowledge, this is the first study elucidating the correlation between *ACE* (I/D) polymorphism and risk and outcome of MM, as well as, with response to bortezomib under *in vitro* conditions. Our findings provide the evidence implicating *ACE* (I/D) polymorphism in the development of MM.

In the present study, an association between *ACE* (I/D) polymorphism and MM risk was observed, where the DD genotype significantly conferred higher disease risk as compared to ID/II genotype. Many data indicate the association between D allele and cancer development, for example Liu et al. has highlighted the correlation between the presence of D allele and the development of moderate-sized adenocarcinomas and metastatic cancer (30). Similar findings were described by Ebert et al. in patients with gastric cancer in German population (31). Furthermore, Zha et al. found that the DD genotypes were associated with increased risk of hepatocellular carcinoma compared with these patients with II genotypes in Chinese Dai population (32).

In the case of hematological malignancies it is suggested, that II genotype can increase the patient's OS above the median depending on the type of leukemia (33). MM is a hematological disorder which arises from proliferation of monoclonal plasma cells derived from B-cells (33). Some leukemias, like CLL or B-ALL (B-cell acute lymphoblastic leukemia) affect the function of B cells. This suggests that in MM patients similar findings might be observed. We have noticed no association between studied genotypes and OS in MM. Moreover, we did not find correlation between *ACE* (I/D) polymorphism and clinical data/laboratory reports.

According to our knowledge, the relationship between *ACE* I/D polymorphism and the response to bortezomib under *in vitro* conditions was not investigated. We originally hypothesized that this polymorphism might interact with bortezomib treatment. Matsuki-Maramoto et al. found that bortezomib treatment might have suppressive effects on the immune and RAAS in NZB/W F1 mice (34). Beside direct effects of angiotensin II, like increasing the volume of fluid and blood pressure, it also regulates cell proliferation and growth. Qin et al. demonstrated that angiotensin II induced the expression of proteasome protein subunits (35). Bortezomib, as a proteasome inhibitor, leads to apoptosis (36). The introduction of bortezomib, as well as the immunomodulatory agents like thalidomide and lenalidomide influenced the survival of MM patients (6, 37). Bortezomib-based chemotherapy is preferred in MM patients with renal failure (22, 36).

Summarizing, we saw no consistent results of *ACE* polymorphism on apoptosis and necrosis in cell cultures derived from MM cases treated with bortezomib. The bone marrow plasma cells may contain somatic mutations in various DNA segments, which may interfere the analysis of *ACE* I/D polymorphism. We found spontaneous apoptosis and necrosis in cultures without borteozmib, which may be caused by laboratory conditions or changes in the microenvironment. Alternatively, myeloma cell lines could be used. However, in the case of studied polymorphism there are not available reference cell lines with all I/D genotypes, which may be used in *in vitro* study.

A limitation of our study is relatively small sample size in part due to the low incidence of the disease. Further analysis on larger cohort can help better understand the significance of *ACE* I/D polymorphism in the pathobiology of MM.

In conclusion, this study found that DD genotype is associated with more than 2-fold higher predisposition to MM. However, we did not confirm the association between studied *ACE* (I/D) polymorphism with disease outcome and bortezomib response under *in vitro* conditions. Our results may help to better understand the role of *ACE* (I/D) polymorphism in the diverse biology of MM.

## Author Contributions

SZ, SP-M, MW-L, ML, SC, IK-P, and JJ carried out the experiment. SZ, SP-M, MW-L, IK-P, and JJ performed the molecular analysis of ACE polymorphism. SC performed cytogenetic analysis. SZ, ML, and SP-M performed *in vitro* study with bortezomib. GS-K, MH, and AF were involved in planning and supervised the work. SZ, A-S-S, MM, and WS processed the experimental data, performed the analysis. SZ, SP-M, and WS designed the figures. SZ, A-S-S, and WS wrote the manuscript with support from GS-K, MH, and AF. All authors discussed the results and commented on the manuscript.

### Conflict of Interest Statement

The authors declare that the research was conducted in the absence of any commercial or financial relationships that could be construed as a potential conflict of interest.
